# A Critical Review of Personal Statements Submitted by Dermatology Residency Applicants

**DOI:** 10.1155/2014/934874

**Published:** 2014-09-14

**Authors:** Jeannette Olazagasti, Farzam Gorouhi, Nasim Fazel

**Affiliations:** ^1^School of Medicine, University of Puerto Rico, San Juan, PR 00936, USA; ^2^University of California, Davis, Sacramento, CA 95816, USA; ^3^Department of Dermatology, University of California, Davis School of Medicine, 3301 C Street, Suite 1400, Sacramento, CA 95816, USA

## Abstract

*Background*. A strong personal statement is deemed favorable in the overall application review process. However, research on the role of personal statements in the application process is lacking.* Objective*. To determine if personal statements from matched applicants differ from unmatched applicants.* Methods*. All dermatology residency applications (*n* = 332) submitted to UC Davis Dermatology in the year of 2012 were evaluated. Two investigators identified the characteristics and recurring themes of content present in the personal statements. Then, both investigators individually evaluated the content of these personal statements in order to determine if any of the defined themes was present. Chi-square, Fisher's exact, and reliability tests were used.* Results*. The following themes were emphasized more often by the matched applicants than the unmatched applicants as their reasons for going into dermatology are to study the cutaneous manifestations of systemic disease (33.8% versus 22.8%), to contribute to the literature gap (8.3% versus 1.1%), and to study the pathophysiology of skin diseases (8.3% versus 2.2%; *P* ≤ 0.05 for all).* Conclusion*. The prevalence of certain themes in personal statements of dermatology applicants differs according to match status; nevertheless, whether certain themes impact match outcome needs to be further elucidated.

## 1. Introduction

Medical students applying for dermatology residency programs submit a less than 2-page personal statement in which they elaborate on themselves and their interest in dermatology.

While the Electronic Residency Application Service (ERAS) facilitates the residency application process as a centralized service that distributes all necessary documents to prospective residency programs including medical school transcripts, USMLE test scores, and letters of recommendation, it has a few shortcomings. First, the applications have become very standardized; therefore the personal statement is the only place the applicants can express their personality and interests. Second, ERAS provides limited instructions for composing the personal statements including allowed size limits and characters [1]. Nevertheless, the American Medical Association (AMA) advises applicants to address three questions: (1) what got you interested in a particular residency? (2) what are you looking for in a residency program? and (3) what are your goals as that specialist? Furthermore, there are numerous residency guides appearing in Google web searches which often include variations of these three questions [1].

Successful matching into a dermatology residency program has become a competitive process [2]. Every year, approximately 500 medical students apply for approximately 370 dermatology residency positions [3]. While prospective candidates believe that a strong personal statement will increase their chances of matching, there is a lack of research on the impact it has on the overall application review and selection process [1]. Nonetheless, according to the “Results of the 2012 National Resident Matching Program (NRMP) Director Survey,” 74% of dermatology program directors responded that the personal statement is more important than USMLE scores or clerkship grades in the residency candidate selection process [4].

We decided to evaluate the personal statements submitted by applicants to a dermatology residency program at a major academic teaching hospital with the objective of determining which themes of content were more frequently emphasized. Furthermore, we sought to investigate if the themes were different between the matched and unmatched groups and whether certain themes had a higher correlation with successful matching.

## 2. Materials and Methods

This study was approved by the University of California, Davis Institutional Review Board as an exemption. All applications (*n* = 332) to the UC Davis Dermatology Residency Program in the year 2012 were analyzed. Essays were deidentified by removing the applicant's name and other identifiable variables and a randomly generated identification number was used to link the essay to other ERAS application data. Reviewers were blinded to the candidate's other application data.

Two investigators (JO and FG) who were blinded to the match outcome initially evaluated 50 randomly selected personal statements in order to identify the characteristics and recurring themes of content. Then, both investigators individually evaluated the content of each of these 50 personal statements in order to determine if any of the defined themes was present. For this initial analysis, the interrater reliability was deemed to be satisfactory (93%) and any disagreements were resolved by consensus. The content of the remaining personal statements was subsequently evaluated by JO. Therefore, all personal statements (*n* = 332) submitted to UC Davis Dermatology Program in the year 2012 were evaluated. Match outcomes of the respective candidates were retrieved from the NRMP website.

Differences in the prevalence of the themes between the matched and unmatched groups were subsequently calculated along with their corresponding 95% confidence intervals (CIs). Chi-square and Fisher's exact tests were used when appropriate in order to assess whether these differences in the prevalence were statistically significant. *P* values equal to or less than 0.05 were considered significant. STATA 12 statistical software (StataCorp LP) was used for this analysis.

## 3. Results

The content of all personal statements (*n* = 332) from the dermatology applicants was evaluated. The initial screening of the 50 randomly selected personal statements resulted in the description of 10 main themes of content, each with its own subdivisions, giving a total of 47 characteristic themes (Table 1). Each theme was defined in a measurable term to minimize interrater variability. Interrater reliability during the initial analysis of 50 randomly was satisfactory (93%), indicating that both investigators agreed with a high level of consistency on whether any of the defined characteristic themes of content was present in a particular personal statement. The prevalence of the 47 characteristic themes reported in the personal statements of matched and unmatched dermatology applicants is shown in Table 1.

The most commonly stated themes in both the matched and unmatched groups were “discussion of a cutaneous disease,” “why dermatology,” and “story telling.” Other specialties have found an increasing number of personal statements sharing common features. Max et al. conducted a study where they evaluated the content of personal statements submitted by anesthesiology residency applicants at a major academic teaching hospital [5]. They found that the personal statement in a typical anesthesiology residency application revolves around one of thirteen common themes [5]. Similarly, in a study examining the personal statements submitted by radiology residency applicants, the statements seemed to consistently mention at least one of eleven defined themes [6]. Therefore, a residency selection committee member recently suggested that statements should be more original and personal since the commonality noted across personal statements limits their utility in distinguishing between candidates who have similar academic records [7].

The prevalence of certain themes found in the statements varied according to whether the applicant successfully matched into dermatology residency or not. For example, personal statements sharing a personal story were less prevalent in the matched group (119/240 (49.6%)) as compared to the unmatched group (55/92 (59.8%)). However, this difference in prevalence did not reach statistical significance (*P* = 0.09). Also discussed less frequently in the matched group versus the unmatched group was the theme of having a family member within the field of medicine (9/240 (3.8%) versus 9/92 (9.8%), *P* = 0.03).

Candidates for dermatology residency positions believe explaining why they chose dermatology is the most important aspect of the personal statement, as this theme was present in about 70% of the submitted statements. Interestingly, a similar observation was also made by Smith et al. They showed that candidates applying to radiology also feel that providing reasons for choosing the field is the most important aspect of the personal statement, since this theme was present in 29 of 30 (96.7%) of the statements [6]. While they did not analyze if these applicants matched or not into radiology residency, they do demonstrate that this theme is of importance for the members of the selection committee since they ranked it the highest out of all the categories of content they defined [6]. While discussing reasons for choosing dermatology was one of the most commonly mentioned themes by residency applicants, these reasons differed significantly between the matched and unmatched applicants. Matched candidates more frequently emphasized their desire to study the cutaneous manifestations of systemic diseases, to contribute to the literature gap, and to understand better the pathophysiology of skin diseases, as their reasons for wanting to go into dermatology. A possible explanation for this finding could be that applicants who successfully match into dermatology often have more extensive research experience and scholar publications. In fact, according to the last “Charting Outcomes in the Match,” matched applicants in dermatology have a mean number of 3.7 research experiences and 7.5 publications, higher than the unmatched applicants who have a mean number of 2.9 and 4.2, respectively [3].

The aforementioned reasons for choosing dermatology of the matched applicants revolved essentially around characteristics specific to the study of dermatology. Interestingly, Max et al. also noticed that personal statements written by anesthesiology applicants tend to be focused about 60% of the time on themes that are specific to the study of anesthesiology, such as interest in physiology and pharmacology [5]. While they did not see if this frequency was different between the matched and unmatched applicants, they did report that stating an interest in the relevant physiology and pharmacology was associated with an invitation to interview at their institution. Their results were unanticipated as they had hypothesized that statements including recurrent, common themes would be viewed less favorably by the selection committee. They thought that reading repeatedly similar subject matter would cause reviewers to view those statements with common themes as less appealing. However, this was not the case since their study showed a strong correlation between the number of common themes in personal statements and an invitation to interview [5].

The idea that personal statements should be more original and personal is supported in different specialties [7–9]. Interestingly, in our study, applicants that successfully matched into dermatology seemed to place less emphasis on a unique storytelling theme or even an applicant-related storyline (Table 1). This was somewhat unexpected considering that the personal statement provides applicants the opportunity to express their personal attributes rather than the explicit details of their CVs and therefore to distinguish themselves from other applicants. According to Smith et al., it is possible that some candidates might have hesitation in sharing their personal qualities for fear of being perceived as conceited [6]. Nonetheless, they believe that this should not be so since their study shows that radiology residency committee members rated the theme of personal attributes highly [6]. It is not feasible to determine, with our current results, if dermatology applicants should in fact try to reveal more of their personalities.

Also less of importance for the matched applicants was to mention if they had a physician relative. This theme was observed significantly less frequently in the statements from the matched group when compared to those from the unmatched group. This falls into accord with the thoughts of residency selection committee members that have an aversion to applicants who sound as if they are going into a specialty just because their parents or family members are in the field. Furthermore, very few applicants (2/332 (0.6%)) admitted that they wanted to go into dermatology for the easy lifestyle. In the past years, residency selection committee members have been trying to differentiate these candidates from the ones that are going into the field because of a genuine desire and passion for the field. Hence, prospective applicants are generally advised not to mention dermatology's lifestyle as their main reason for applying to it. Nevertheless, one of the two applicants who mentioned this theme in our study successfully matched.

A significant limitation of our study was that the personal statements of applicants applying to a single program (UC Davis) were analyzed. Therefore, further research is needed to determine whether or not these results are generalizable to all dermatology residency applicants. While it is possible that our results remain specific only to those applying for a dermatology residency at our institution, it is rather unlikely since the 332 applications reviewed represent 65.4% of the total national pool of applicants to PGY-2 dermatology residencies in 2012 [10]. Another limitation is that the analysis of each personal statement is inevitably subjective; however, the initial analysis of 50 randomly selected personal statements showed that both reviewers showed strong interrater reliability in their assessments. We believe that the personal statement has an important role in the initial residency application screening process. However, its ultimate impact on successful matching into dermatology residency was not investigated, which is a significant limitation of our study. In addition, the role of other factors such as volunteerism and community service in the residency screening process was not explored in this study and would be worthy of further investigation.

The available literature on the topic suggests that the value placed on personal statements might vary depending on the specialty. Crane and Ferraro found that personal statements were the least important factor for selecting emergency medicine residents [11]. Similarly, a study by Taylor et al. showed that obstetrics and gynecology directors ranked personal statements last in importance for interview invitation [12]. The latter study also presented that family practice program directors consider them the second most important factor, implying that different specialties have differing views on the overall significance of personal statements [12]. Nevertheless, the fact that a personal statement's content correlates with clinical aspects of training, as shown by Ferguson et al., suggests that these could potentially be of value for all specialties [13]. Ferguson et al. compared the impact of grades, personal statements, and letters of recommendation to predict performance over the five years of a medical degree and found that personal statements with greater number of common themes were reliable predictors of positive clinical performance [13].

In spite of the research done so far in the field, there is sparse evidence to help medical students and more often than not they still agonize on what they should write in their statements. We understand that our study is the first attempt to analyze the contents of personal statements submitted by dermatology applicants in order to instigate if there are characteristics in them that increase the chance of matching. We impart our results not only to inform students and faculty involved in the match process of what are the trends seen in the personal statements of dermatology applicants, but also to stimulate continued research on this important subject.

## 4. Conclusion

Personal statements of dermatology applicants discuss a number of common, repeated themes. The prevalence of certain themes differs according to whether the applicant successfully matched into residency or not. For example, describing why they chose dermatology was more commonly recognized in the statements of the matched group. On the other hand, stating a personal story was more frequently observed in those of the unmatched group. However, the possibility that describing certain themes in personal statements impacts match outcome is currently under investigation and needs to be further elucidated.

## Figures and Tables

**Table 1:** Characteristic themes of content that appeared in the personal statements.

Characteristic	Prevalence, %		Difference		
	Matched	Not matched	%	95% CI	*P* value
(1) Story telling	64.58	72.83	−8.24	(−19.16, 2.68)	0.1533
Personal story	49.58	59.78	−10.20	(−22.05, 1.65)	0.0958
Case presentation	39.58	32.61	6.97	(−4.43, 18.38)	0.2404
Personal illness	15.83	16.30	−0.471	(−9.32, 8.34)	0.9165
Family illness	15.00	14.13	0.869	(−7.56, 9.30)	0.8415
Family relevance to dermatology	5.83	5.43	0.399	(−5.10, 5.90)	0.8887
Family relevance to medicine	3.75	9.78	−6.03	(−12.56, 0.496)	0.0298
(2) Why medicine?	17.50	19.57	−2.07	(−11.49, 7.36)	0.6616
(3) Why dermatology?	75.42	69.57	5.85	(−5.01, 16.72)	0.2779
Multidisciplinary	34.17	32.61	1.56	(−9.75, 12.86)	0.7881
Visual	24.58	19.57	5.02	(−4.75, 14.78)	0.3322
Cutaneous manifestations of systemic disease	33.75	22.83	10.92	(0.467, 21.38)	0.0535
Social/psychosocial issues	39.17	35.87	3.30	(−8.29, 14.88)	0.5801
Chronicity of disease	7.50	8.70	−1.20	(−7.85, 5.46)	0.7167
Literature gap	8.33	1.09	7.25	(3.16, 11.33)	0.0152
Complexity of the field	19.17	19.57	−0.398	(−9.91, 9.12)	0.9343
Technology	5.00	4.35	0.652	(−4.34, 5.65)	0.8039
Patients of all ages	10.00	8.70	1.30	(−5.59, 8.20)	0.7185
Better understanding of the pathophysiology of skin disease	8.33	2.17	6.16	(1.57, 10.75)	0.0434
Lifestyle	0.417	1.09	−0.670	(−2.94, 1.60)	0.4799
(4) Accomplishment	61.25	56.52	4.73	(−7.13, 16.59)	0.4313
Fellowship/research	62.08	58.70	3.39	(−8.40, 15.17)	0.5709
Publications	25.00	23.91	1.09	(−9.21, 11.38)	0.8371
Volunteer service	32.50	25.00	7.50	(−3.15, 18.15)	0.1837
Skin cancer screenings	7.92	6.52	1.39	(−4.70, 7.49)	0.6664
Leadership	10.00	10.87	−0.870	(−8.28, 6.54)	0.8151
(5) Quotation	23.75	25.00	−1.25	(−11.61, 9.11)	0.8116
By famous figures	4.17	7.61	−3.44	(−9.42, 2.54)	0.2028
Other or self-quotation	12.92	14.13	−1.21	(−9.50, 7.07)	0.7703
(6) Career goal	51.25	47.83	3.42	(−8.58, 15.43)	0.5765
Purely clinician	4.17	5.43	−1.27	(−6.55, 4.01)	0.6185
Physician scientist	36.67	32.61	4.06	(−7.30, 15.41)	0.4893
Physician educator	22.50	21.74	0.761	(−9.19, 10.71)	0.8815
(7) Subspecialty within dermatology	12.92	16.30	−3.39	(−12.05, 5.27)	0.4239
Procedural dermatology/MOHs micrographic surgery	2.92	3.26	−0.344	(−4.55, 3.86)	0.8695
Pediatric dermatology	5.42	6.52	−1.11	(−6.90, 4.70)	0.6980
Dermatopathology	2.08	3.26	−1.18	(−5.23, 2.88)	0.5312
Immunodermatology	2.50	3.26	−0.761	(−4.89, 3.37)	0.7024
(8) Name a cutaneous disease	76.25	71.74	7.79	(−0.798, 16.38)	0.1031
Mentions > 5	20.83	13.04	4.51	(−6.15, 15.17)	0.3956
(9) Significant other	2.50	3.26	−7.61	(−4.89, 3.37)	0.7024
(10) Personalized application	13.75	8.70	5.05	(−2.17, 14.45)	0.2103
Interest in California	5.00	3.26	1.74	(−2.82, 6.30)	0.4947
Interest in UC Davis	8.75	6.52	2.23	(−3.96, 8.41)	0.5062
Mentions UC Davis Faculty	1.25	2.17	−0.924	(−4.22, 2.37)	0.5362
Interest in other institutions	25.00	30.43	−5.43	(−16.32, 5.45)	0.3153
Mentions the faculty of other institutions	29.17	29.35	−0.181	(−11.12, 10.76)	0.9741
